# SG-APSIC1146: Reducing bacterial contamination in the dental unit waterline (DUWL) in dental clinics

**DOI:** 10.1017/ash.2023.68

**Published:** 2023-03-16

**Authors:** Lily Lang, Priscilla Chao Jang Shing, Kenneth Low Meng Tze

**Affiliations:** National Healthcare Group Polyclinics, Singapore; Singapore National Healthcare Group Polyclinics, Singapore; Singapore National Healthcare Group Polyclinics, Singapore

## Abstract

**Objectives:** We evaluated the effectiveness of using appropriate chemical(s) to treat the dental unit waterline (DUWL), and we recommended appropriate strategies to manage the DUWL system to maintain bacteria concentration below minimum recommended levels. **Methods:** Initial water samples were collected aseptically from the handpieces of the DUWL in dental clinics to assess the bacterial load prior to treatment of the dental unit. The dental staff were educated on the management and treatment of the DUWL. Appropriate chemicals were introduced to the DUWL system. Following the treatment, samples of water from the DUWLs were collected to assess the bacterial load. **Results:** The US CDC recommends a safe level of bacterial load of <500 CFU per mL of heterotrophic bacteria in the standard for drinking water by the US EPA. Initial results for the DUWL water showed unacceptably high levels of bacterial load between 1,930 and 35,000 CFU per mL prior to treatment. Subsequent sampling of DUWL water with treatment of appropriate chemicals showed vast reductions of the bacterial loads in all the dental units, with bacterial counts between <1 and 72 CFU per mL. **Conclusions:** It is important to ensure ongoing education and regular treatment with appropriate chemical and effective management and monitoring of all DUWLs from dental chairs to ensure that the water produced meets safe drinking standards.

